# Integrated treatment utilizing both Chinese and Western medicine for refractory diabetic foot ulcers: a case report

**DOI:** 10.3389/fendo.2025.1704770

**Published:** 2025-11-05

**Authors:** Luyao Wang, Changting Sheng, Jiaxin Liu, Rong Chen, Rensong Yue, Maoyi Yang, Zhipeng Hu

**Affiliations:** ^1^ Department of Endocrinology,Hospital of Chengdu University of Traditional Chinese Medicine, Chengdu, China; ^2^ School of Medicine, Qinghai University, Xining, China

**Keywords:** diabetic foot ulcers, traditional Chinese Medicine, wound, wound healing, wound care

## Abstract

Diabetic foot ulcer (DFU) is a severe complication of diabetes associated with a high risk of amputation and long-term disability. We report the case of a 57-year-old man with a Wagner grade IV DFU whose condition continued to deteriorate despite two toe amputations. An integrative treatment strategy combining traditional Chinese medicine (TCM) and western medicine was subsequently employed and proved effective. The TCM regimen included oral decoctions and topical herbal applications, while the western medical approach encompassed targeted therapies including glycemic control, anti-infective therapy, circulatory enhancement, anticoagulation, neurotrophic support, and nutritional management. Over a 144-day course, the ulcer demonstrated near-complete closure at discharge, with full epithelialization and functional recovery confirmed at follow-up. HbA1c decreased from 13.5% to 7.0%, indicating improved metabolic control. This case illustrates that an integrative management strategy can effectively control infection, promote tissue regeneration, and restore limb function in advanced DFU.

## Introduction

According to the International Diabetes Federation (IDF) Diabetes Atlas, approximately 500 million adults globally currently suffer from diabetes mellitus (DM), and this number is expected to increase by 46% by 2045 (1). Diabetic foot ulcers (DFUs) are one of the most severe complications of DM, affecting 6.3% of patients (2), and a DM patient undergoes an amputation due to DFU every 20 seconds (3). DFUs significantly contribute to disability and infection-related mortality among DM patients. They often occur in the distal extremities and are commonly associated with neuropathy and peripheral vascular disease, leading to infections, ulcer formation, and/or deep tissue damage. Local tissue infection, peripheral neuropathy, and peripheral arterial disease are key risk factors influencing DFU onset (4–8).

Current clinical treatments involve systemic adjustments (such as blood sugar control, anti-infection measures, improved microcirculation, anticoagulation, and nerve nutrition) as well as wound management strategies (including debridement and repair techniques like skin grafting, stem cell therapy, and phototherapy) (9–17). In recent years, the Ilizarov method has also been employed to enhance local blood flow and promote ulcer healing in refractory DFU cases, particularly when conventional therapies fail (18–20). However, its application is often limited by technical complexity and patient tolerance, and managing high-grade DFUs complicated by comorbidities remains a major clinical challenge in achieving wound healing and limb preservation (21, 22).

TCM categorizes DFUs under gangrene and boasts a history of substantial clinical experience in treating these conditions. TCM treatments can be classified into internal and external therapies, renowned for their diverse approaches and effectiveness in promoting skin healing. TCM addresses deficiency (“xu”), stasis (“yu”), evil influences (“xie”), and decay (“fu”), offering targeted therapies to accelerate wound healing and reduce recurrence. Modern medical research has evidenced TCM’s significant efficacy in DFU treatment by exploring cells, proteins, molecules, and signaling pathways (23–27). This case report describes the successful treatment of a patient with a Wagner grade IV DFU and multiple comorbidities using an integrative regimen combining TCM and western medicine. To our knowledge, few detailed cases have documented complete healing of such advanced DFUs with integrative therapy, highlighting its potential value in complex diabetic wound management.

## Case report

A 57-year-old male patient was admitted on April 28, 2023, with a five-year history of elevated blood sugar and a one-week history of a skin ulcer on the second toe and sole of his left foot. He was diagnosed with Type 2 diabetes five years ago, with a peak blood glucose level of 20 mmol/L, and began treatment with metformin, glimepiride, and gliclazide. However, he did not consistently monitor his blood sugar levels or adhere to his oral hypoglycemic medication regimen. One week prior to admission, he observed redness, swelling, numbness, and pain in his left foot, followed by ulceration on the second and third toes and the sole, emitting a pale yellow purulent discharge. The skin was dark and emitted a foul odor. He sought medical attention at Deyang People’s Hospital, where the third toe of his left foot was amputated. Post-operative care focused on infection control, boosting circulation, and debridement. A CT scan of the left foot indicated the absence of the third toe, slightly decreased bone density at the distal ends of the second to fourth metatarsals, and swelling in the surrounding soft tissues. Irregularities were noted at the proximal end of the left first metatarsal, likely due to degeneration or a previous injury. Three days ago, his wound ulcers worsened; the second toe became necrotic and black, with persistent pain, redness, and swelling, prompting treatment at our hospital. He reported numbness and pain in the left foot, dry mouth with excessive thirst, a bitter taste, frequent urination, twice-nightly nocturia, and sticky stools. Clinical examination revealed a yellow, greasy tongue coating, a dark red tongue body, and a slippery, rapid pulse.

## Physical examination

**Figure 1:**
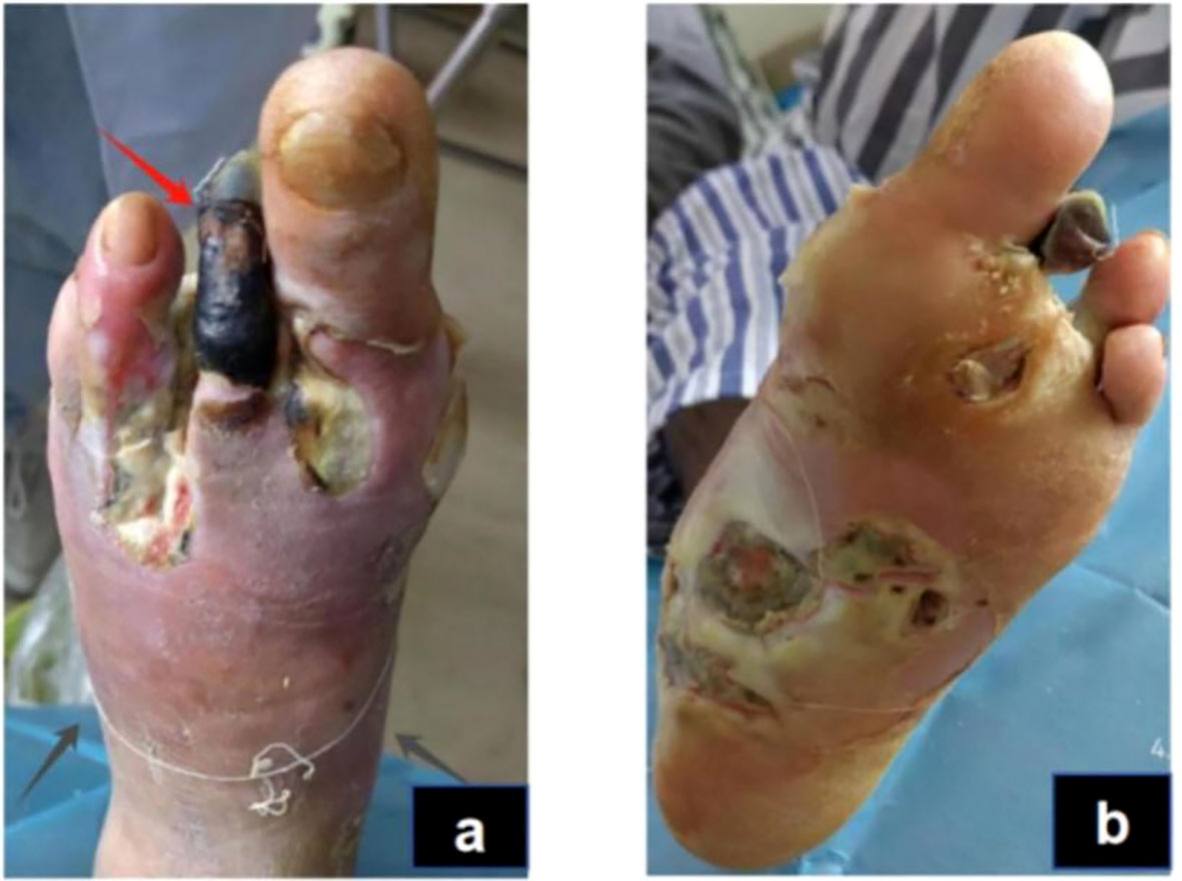
Acute infection phase (Day 1): The red arrow indicates gangrene of the second toe of the left foot,and the black arrows show the necrotic boundary of the foot **(A)**. The redness and swelling of the sole are obvious, and the boundary of necrosis is unclear **(B)**.

The physical examination of the patient revealed a body temperature of 36.3°C, a pulse rate of 74 beats per minute, a respiratory rate of 20 breaths per minute, and blood pressure of 135/79 mmHg. The patient was conscious but displayed depressive symptoms. Cardiac, pulmonary, and abdominal assessments showed no significant abnormalities. Mild pitting edema was noted in both lower limbs, with non-palpable dorsal pedal and posterior tibial arteries bilaterally. The right foot had intact skin but a slightly reduced skin temperature. Conversely, the left foot presented with a lower skin temperature, absence of the third toe, and multiple ulcerations on the second toe and sole; the second toe appeared necrotic. The ulcerated area on the dorsum of the foot measured approximately 3 x 4 cm, while ulcerations on the sole reached up to 10 x 12 cm. The wound was red and discharged a white secretion with a foul odor (**Figure 1**).

## Laboratory tests

Upon admission, the patient exhibited poor metabolic control (HbA1c 13.5%, fasting plasma glucose 30.66 mmol/L), systemic inflammation (CRP 91.86 mg/L, WBC 9.82 × 10^9^/L), and hypoproteinemia (albumin 28.3 g/L).The results of all laboratory examinations are summarized in **Table 1**. A bilateral lower limb vascular ultrasound revealed plaque formation in both common femoral arteries, varicose veins in the left great saphenous vein, a hypoechoic nodule in the left popliteal area, and enlarged lymph nodes in the left inguinal region. The chest and foot CT scans indicated several findings (1): pulmonary emphysema and pulmonary bullae in the upper lobes of both lungs, with slight interstitial changes and scattered small fibrous stripes (2); scattered small solid and ground-glass nodules in both lungs, considered low-risk, with an annual review recommended (3); slight coronary artery calcification (4); small amounts of pleural effusion on both sides (5); extensive bone absorption and destruction in the metatarsal and phalangeal bones of the left foot, most notably in the distal fourth metatarsal, with loss of joint structures, widespread swelling of the surrounding soft tissue accompanied by scattered air, and partial discontinuity of the skin, suggesting the possibility of a diabetic foot; clinical correlation is advised. The wound pathogen culture identified infections with Candida parapsilosis and Enterococcus faecium, which are sensitive to levofloxacin, gentamicin, ampicillin, ciprofloxacin, linezolid, and penicillin, but highly resistant to streptomycin, erythromycin, and tetracycline.

**Table 1:** The laboratory examination results of the patient.

Laboratory examination results	Day 1	Day 14	Day 25	Day 43	Day 75	Day 115	Day 144
White blood cell count (10^9^/L)	9.82	7.04	2.47	4.24	3.98	4.62	4.76
Neutrophil counts(10^9^/L)	8.86	5.81	1.89	3.42	3.35	2.74	3.02
Red blood cell count (10^9^/L)	3.11	2.86	2.64	3.45	4.48	4.57	4.62
Hemoglobin(g/L)	97	89	81	106	128	135	141
C-reactive protein(mg/L)	91.86	13.34	64.33	2.45	1.97	1.3	0
K+(mmol/L)	3.99	4.17	3.21	4.5	4.17	4.68	4.14
Total Protein(g/L)	52	55.4	51.7	60.6	63.1	66.4	68
Albumin(g/L)	28.3	31.1	29.4	35.3	38.1	40.2	41.9
Creatinine(umol/L)	55.1	59.5	50.9	59.5	62.6	67.4	62
Uric Acid(umol/L)	118	167	142	214	230	279	262
HbA _1c_(%)	13.5	–	–	–	–	7	–
fasting blood glucose (mmol/L)	30.66	9.94	16.46	11.86	8.87	7.57	5.11
Wound secretion culture	Candida parapsilosis,Enterococcus faecalis.Sensitive to gentamicin 120, ampicillin, ciprofloxacin, levofloxacin, linezolid, etc., and highly resistant to streptomycin, erythromycin, and tetracycline.	Candida albicans,Staphylococcus lugdunensis.Sensitive to amphotericin B, fluconazole, ciprofloxacin, clindamycin, gentamicin, levofloxacin, linezolid, etc., and resistant to oxacillin and penicillin.	Candida albicans is sensitive to amphotericin B, fluconazole, voriconazole, caspofungin, and micafungin.	no bacteria cultured	no bacteria cultured	no bacteria cultured	no bacteria cultured

## Admission diagnoses

1. Type 2 diabetic foot disease; 2. Type 2 diabetes with multiple complications; 3. Lower limb skin infection; 4. Hypoproteinemia; 5. Protein-energy malnutrition; 6. Moderate anemia; 7. Emphysema.

## Course of diagnosis and treatment

Given the patient’s severe infection, compromised baseline health, and significant clinical fluctuations, we implemented a staged treatment strategy.

### Acute infection phase (days 1-42)

The acute infection phase was defined in this case as the period showing active local necrosis, purulent discharge, and elevated inflammatory markers (e.g., CRP >10 mg/L or WBC >9×10^9^/L), consistent with the clinical manifestations of moderate-to-severe diabetic foot infection as described in the IWGDF guidelines (28). Upon admission, the patient received symptomatic treatments including glucose control, anti-infection measures, circulation support, anticoagulation, neuro-nutrition, and nutritional management (**Table 2**).

**Table 2:** The Western medicine treatment of the patient.

Treatment	Measures	Usage and dosage
Blood sugar control	Metformin Hydrochloride Tablets;Insulin Glargine	0.5g,oral administration,ter in die,1-144d;14 iu of insulin subcutaneous injection before bedtime,1-144d.
Anti-infection therapy	Latamoxef Sodium;Ornidazole;levofloxacin;Cefuroxime sodium;Piperacillin and Tazobactam	1g, intravenous drip, quaque 12 hours,1-18d,28-42d;0.5g, intravenous drip,quaque 12 hours,1-24d;0.5g, intravenous drip,quaque die,18-24d;1.5g, intravenous drip, quaque 8 hours,25-26d;4.5g, intravenous drip, quaque 8 hours,26-27d.
Improve circulation	Alprostadil Injection	20ug,intravenous drip,quaque die,1-144d.
Anticoagulation	Enoxaparin sodium Injection	40mg,intravenous drip,quaque die,1-144d.
Nutrition of the nervous system	Thioctic Acid Injection	0.6g,intravenous drip,quaque die,1-144d.
nutrition management	Human Albumin Injection	10g,intravenous drip,quaque die,3-20d.
lower body temperature	Bupleurum Injection	2ml,intramuscular injection,quaque die,15-20d.
potassium supplement	Potassium Chloride Sustained-release Tablets	1g,oral administration,ter in die,15-25d.

Antimicrobial therapy was dynamically adjusted based on wound culture and drug-sensitivity results. Empiric broad-spectrum coverage with latamoxef sodium (Days 1–18) and ornidazole (Days 1–24) was initiated to control mixed aerobic–anaerobic infection. After Candida parapsilosis and Enterococcus faecalis (fluoroquinolone-sensitive) were identified on Day 1, the regimen was de-escalated to levofloxacin (Days 18–24) while continuing ornidazole. Later cultures on Days 14 and 25 revealed Candida albicans and Staphylococcus lugdunensis. When systemic deterioration and CRP elevation occurred on Day 25, therapy was temporarily escalated to cefuroxime sodium (Days 25–26) and piperacillin–tazobactam (Days 26–27) for broader Gram-positive and Gram-negative coverage. After stabilization and ICU discharge, the patient was stepped down to latamoxef sodium (Days 28–42) for consolidation. From Day 43 onward, serial cultures were negative, inflammatory markers normalized, and systemic antibiotics were discontinued. Candida species were regarded as surface colonizers; thus, no systemic antifungal therapy was required (**Table 2**).

**Figure 2:**
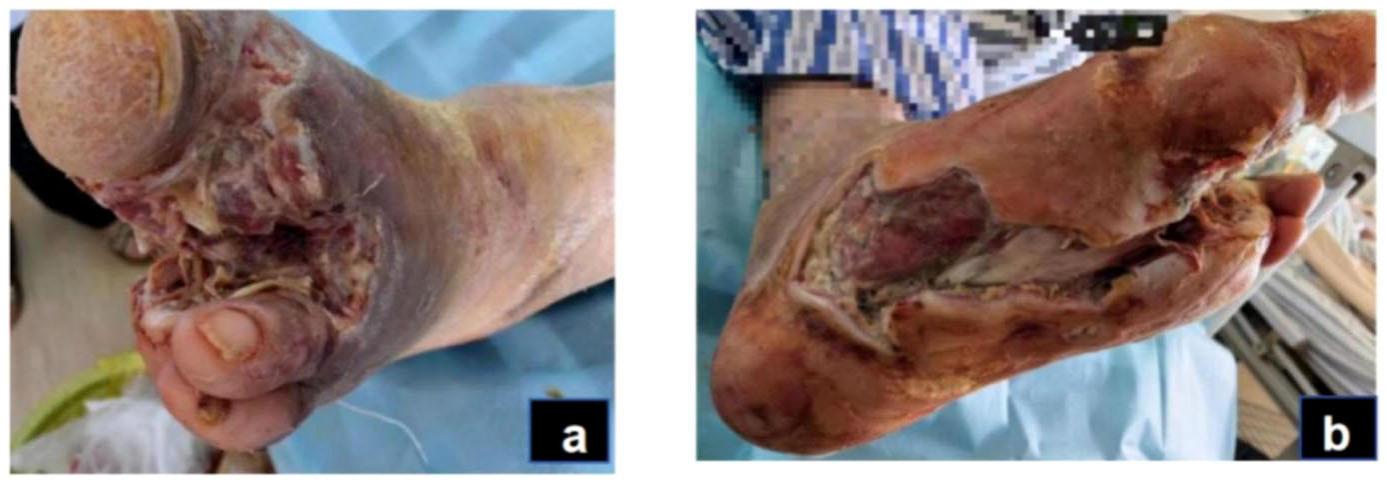
Acute infection phase (day 14): After the patient underwent amputation of the second toe on the left foot **(A)**. Debridement of plantar wound after amputation **(B)**.

For wound care, we disinfected the wound with iodine from the periphery to the center within a 15 cm diameter, followed by conservative sharp debridement of necrotic tissue and iodine with saline irrigation of the wound and sinus tract, then covered with sterile gauze. Dressing changes were conducted every morning. After local redness and swelling diminished, amputation of the second toe of the left foot, along with debridement, was performed on day 14 (**Figure 2**), followed by daily debridement and dressing changes post-surgery. The patient experienced recurrent fevers, peaking at 38.9°C, treated with cooling and potassium replacement therapy. On day 25, the patient suffered respiratory distress, with blood oxygen saturation under a mask oxygenation of only 82-89%. CT scans and bedside ultrasonography indicated significant bilateral pleural effusion, prompting an ICU transfer for further care, including bilateral thoracic puncture and catheter drainage. Pleural fluid analysis showed no significant abnormalities, and the drainage tubes were removed once vital signs stabilized on day 27, allowing the patient to return to our department. Concurrently, a nutritional consultation crafted a weekly diet plan aimed at providing 1.25-1.5 g/kg of high-quality protein per day, with additional supplements of 17–30 g of arginine, 0.57 g/kg of glutamine, and 70–90 mg of vitamin C, recommending short-term omega-3 fatty acid supplementation to enhance nutritional status. Laboratory examinations were conducted at clinically indicated milestones rather than predetermined intervals. During the acute infection period, tests were repeated when the patient’s condition fluctuated (e.g., fever, elevated CRP, or treatment adjustment). After Day 27, when vital signs and wound condition stabilized, laboratory testing frequency was reduced to once every two to three weeks until Day 43, in line with routine clinical practice for stable post-infectious patients.

### Wound healing period (days 43-144):inflammatory phase (days 43-75)

**Figure 3:**
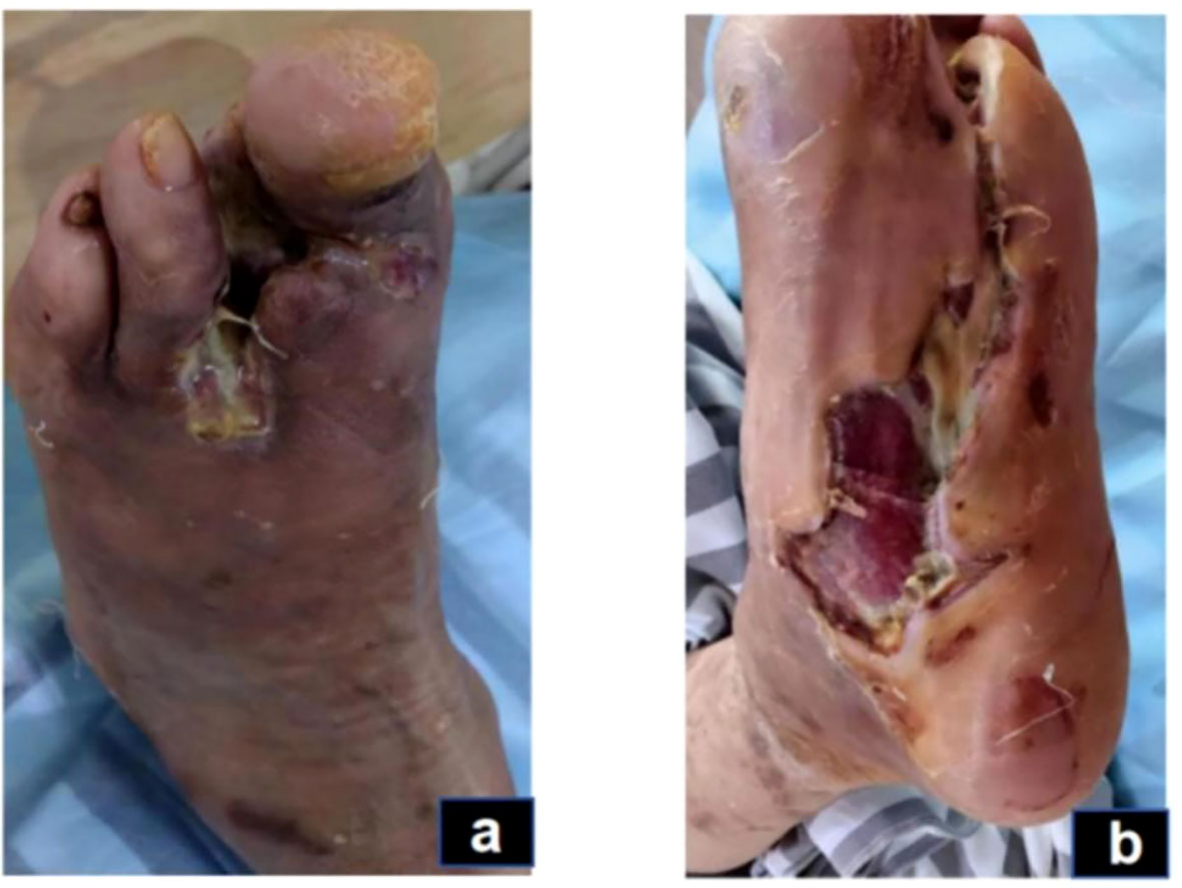
Inflammatory phase (day 43): The swelling on the dorsum of the foot has subsided, but there is still a small amount of secretion **(A)**. The dorsal foot wound has decreased in size, and the wound has shrunk **(B)**.

In this case, the inflammatory phase was characterized by local redness, swelling, necrotic wound edges, and purulent discharge, while systemic infection was largely controlled. These manifestations corresponded to the TCM pattern of Damp-Heat Toxin Syndrome (29, 30).The wound presented abundant exudation and malodor, accompanied by a greasy yellow tongue coating, dark-red tongue body, and slippery rapid pulse—features indicative of this syndrome (**Figure 3**). Treatment during this stage focused on clearing heat, resolving dampness, and detoxifying. A modified Simiao Yong’an Decoction (SYD) was prescribed (Radix Scrophulariae, 30g; Flos Lonicerae Japonicae, 30g; Radix Angelicae Sinensis, 20g; Radix et Rhizoma Glycyrrhizae, 10g; Rhizoma Coptidis, 20g; Radix Scutellariae, 20g; Cortex Phellodendri Chinensis, 20g; Fructus Gardeniae, 20g), boiled into a 400 ml decoction. The patient ingests 100 ml orally 30 minutes post-breakfast and dinner, each dose covering two days. For wound care, cleaning with saline-soaked gauze precedes the application of Moist exposed burn ointment (MEBO) (produced by Shantou Meibo Pharmaceutical Co., Ltd., National Drug Approval Number Z20000004) on the wound, spread 0.5 to 0.8 cm thick with a disposable tongue depressor, and bandaged with sterile gauze. Dressing changes are performed every morning. By day 75, foot swelling subsided, leaving minimal exudate, and surface necrotic tissue was entirely removed, advancing the wound from the inflammatory to the regenerative and remodeling phase.

### Granulation and re-epithelialization phase (days 76-144)

**Figure 4:**
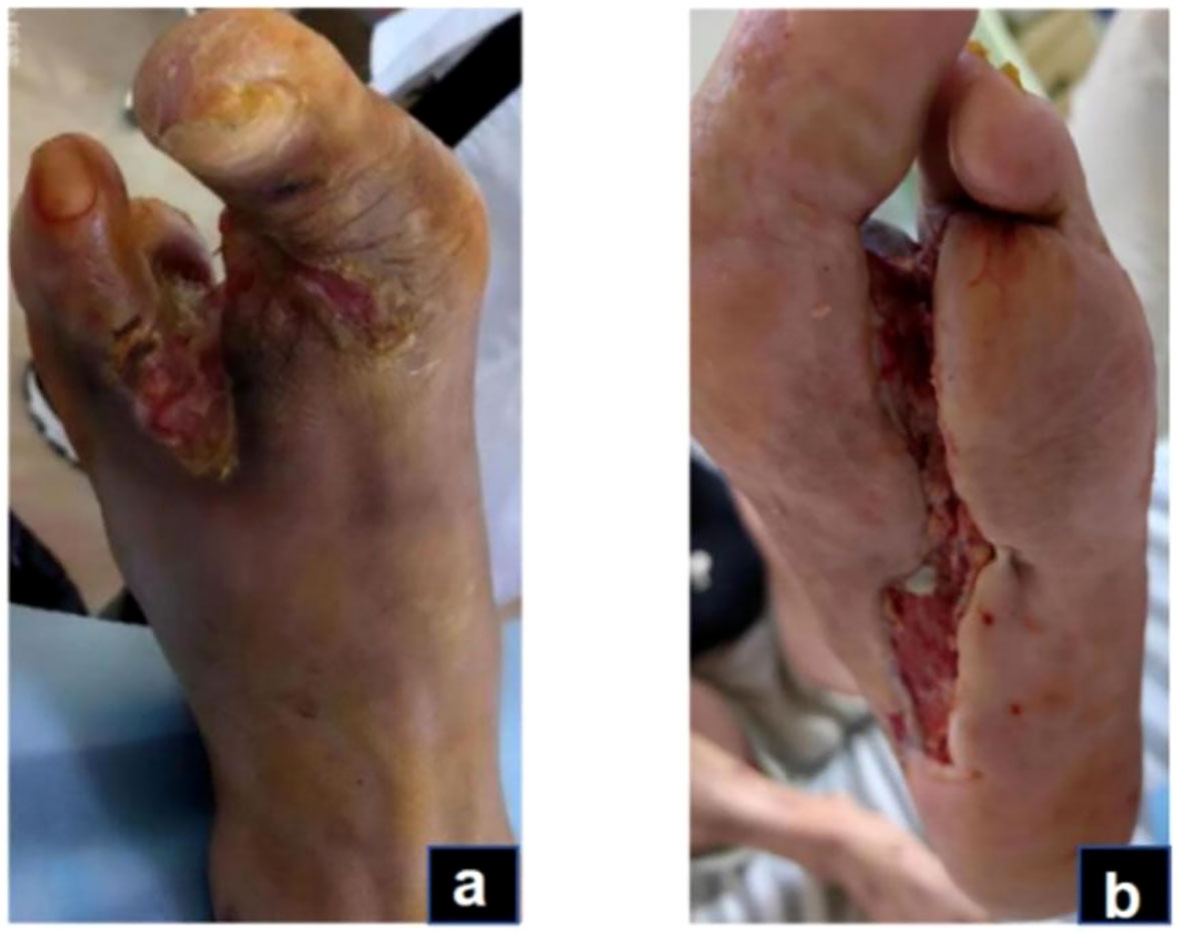
Granulation and re-epithelialization phase (Day 76): The granulation tissue on the dorsum and plantar of the foot grew well **(A, B)**.

**Figure 5:**
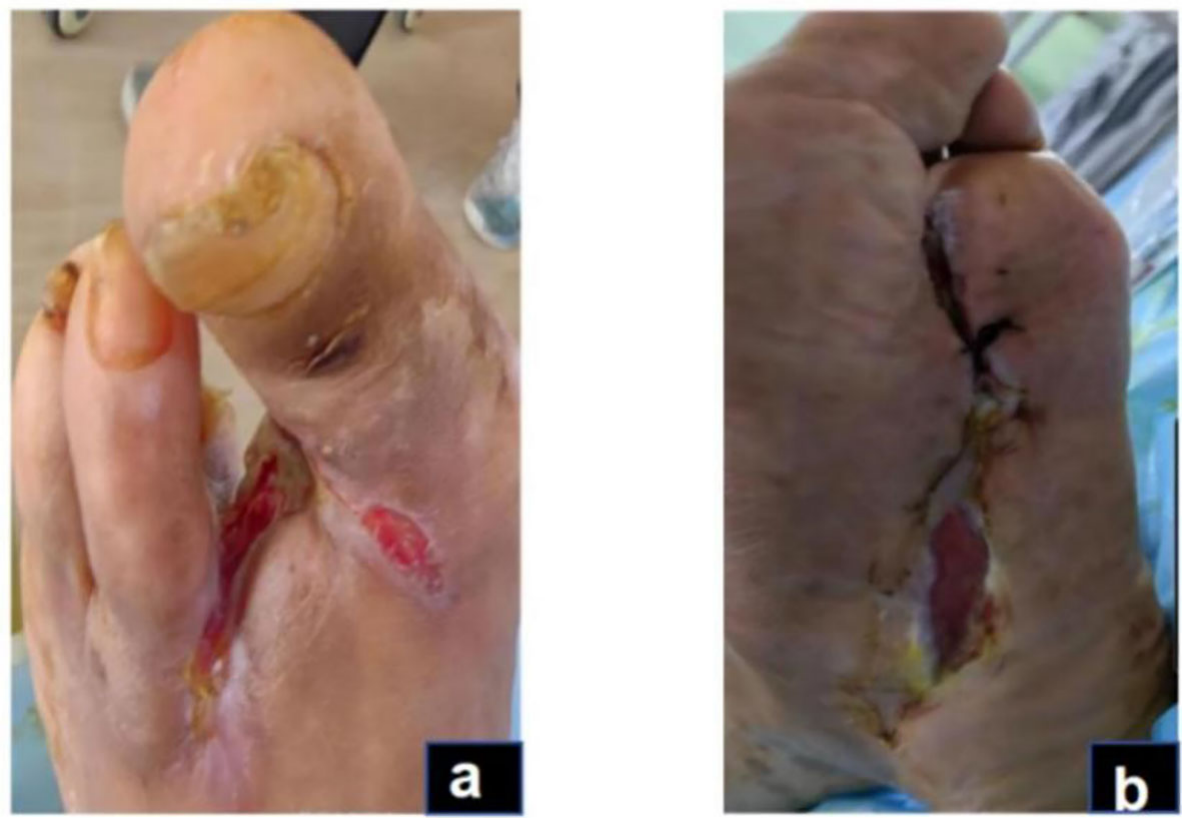
Granulation and re-epithelialization phase (Day 115): The dorsal and plantar wounds of the foot after wound closure of the plantar surface **(A, B)**.

**Figure 6:**
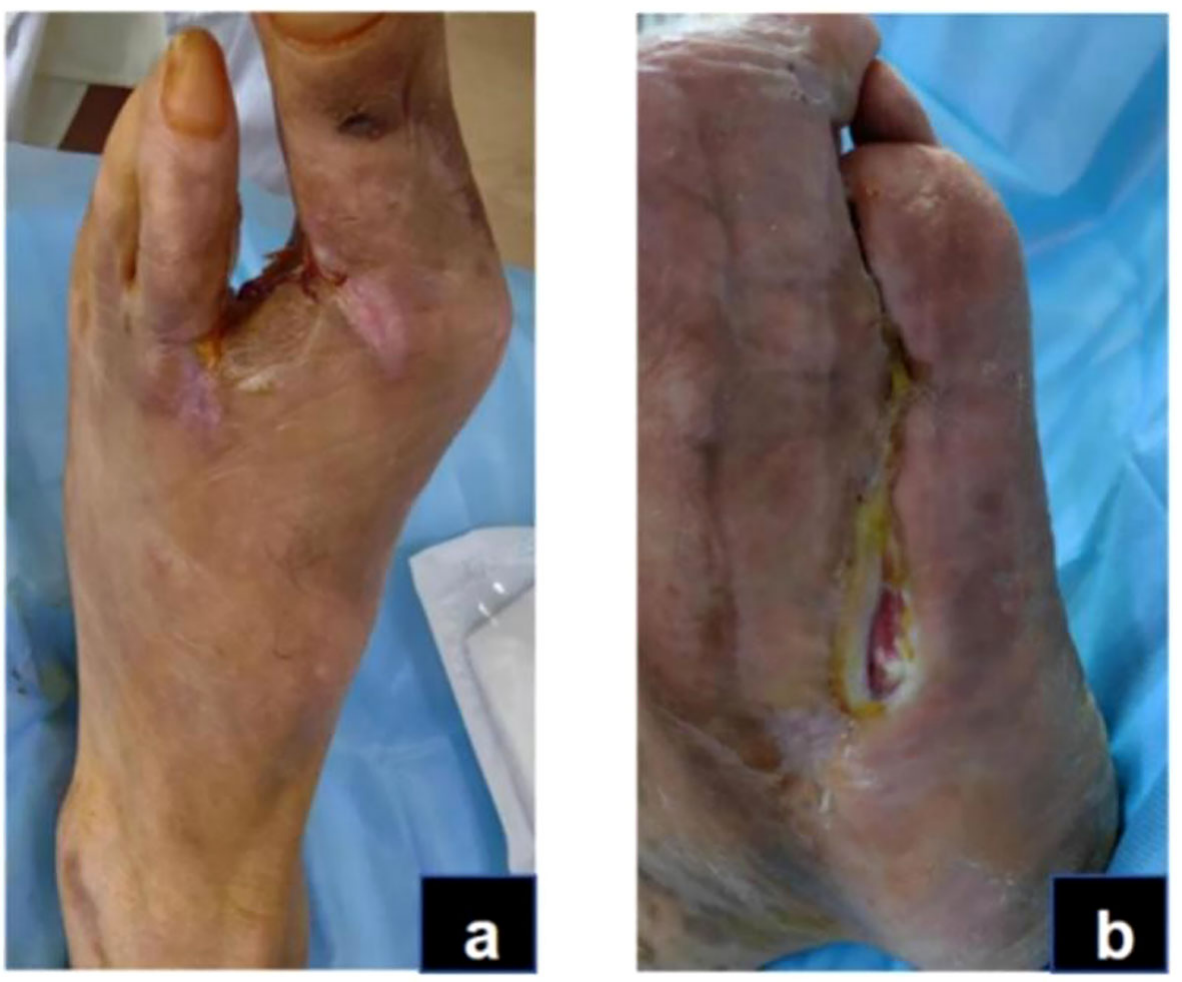
Granulation and re-epithelialization phase (Day 144): The dorsal foot wound healed completely **(A)**. The area of the plantar wound was reduced to 0.5*2.5cm. **(B)**.

**Figure 7:**
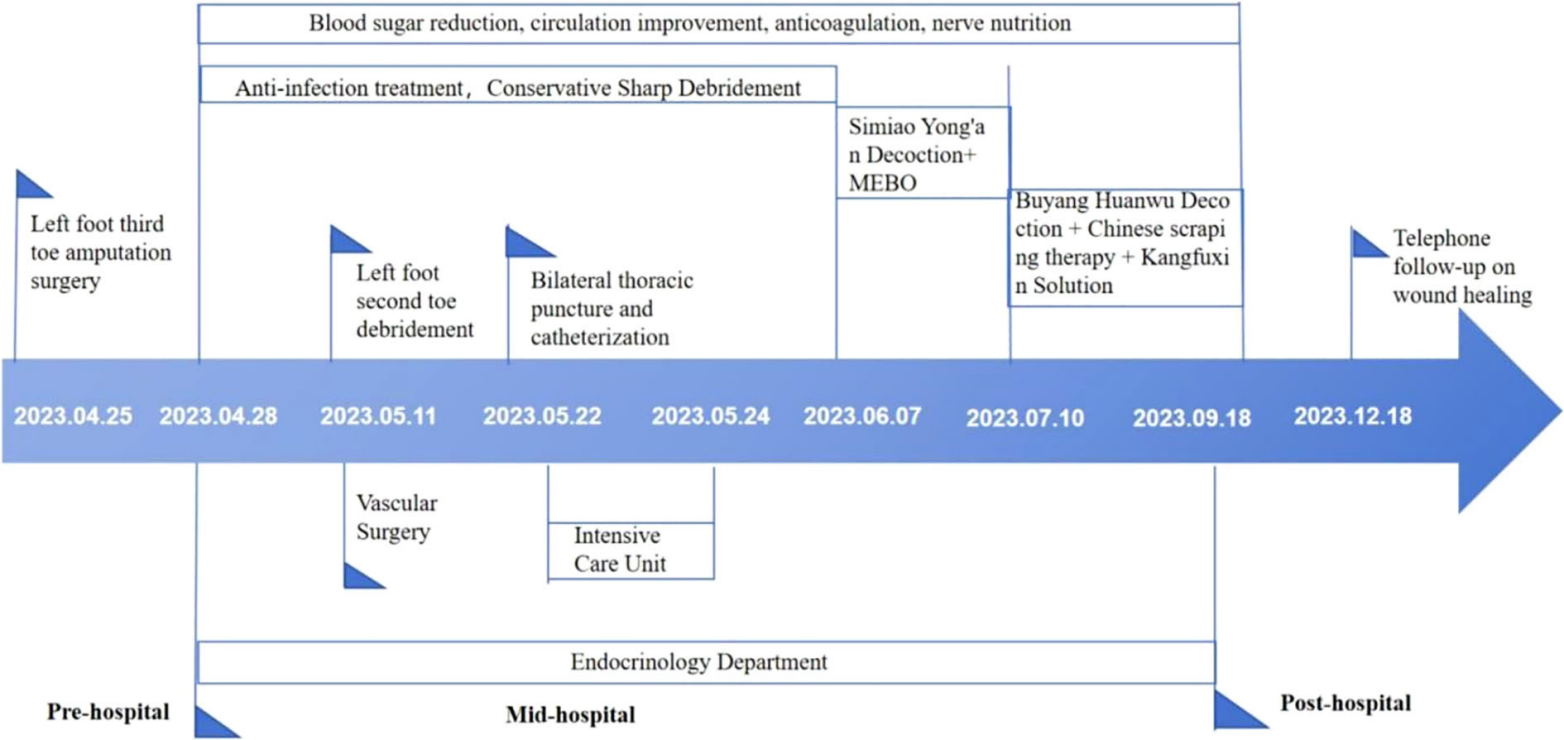
The timeline of the medication process of the patient.

In this case, the transition from the inflammatory to the remodeling phase was defined clinically when exudation and necrosis subsided, granulation tissue became dominant, and inflammatory markers such as CRP had already normalized. These criteria were based on the clinical features described in current wound-healing guidelines and literature (31, 32). During this stage, granulation tissue on both the sole and dorsum of the foot proliferated rapidly, filling the wound bases (**Figure 4**). Due to pathogenic impact, the patient exhibited significant Qi and blood depletion, fatigue, weakness, and a pale complexion with watery discharge, a pale red tongue, white coating, and a deep fine pulse, diagnosed as Qi and blood deficiency syndrome. Treatment involves supporting Qi, invigorating blood, and promoting muscle growth with Buyang Huanwu Decoction (BHD) (Radix Astragali, 20g; Radix Angelicae Sinensis, 15g; Radix Paeoniae Rubra, 15g; Flos Carthami, 15g; Semen Persicae, 15g; Rhizoma Chuanxiong, 15g; Pheretima, 10g). The administration mirrors prior methods. For wound care, cleaning with gauze soaked in KangFuXin Solution (KFXS) (manufactured by Sichuan Good Doctor Panxi Pharmaceutical Co., Ltd., National Drug Approval Number Z51021834) precedes KangFuXin-soaked gauze as an outer dressing, bandaged with sterile materials. Daily dressing changes occur in the morning. By day 115, the granulation tissue largely filled the sole wound. However, the wound size impeded epithelial migration, necessitating sole wound suturing (**Figure 5**) to expedite re-epithelialization. Post-surgery, the wound size reduced to 0.5 x 2.5 cm on the sole, and the dorsum wound fully healed (**Figure 6**). The patient requested discharge, advised to continue oral BHD and topical KFXS usage until complete wound healing. (**Figure 7**).

## Discussion

The wound healing process is typically divided into four continuous and overlapping stages: hemostasis, inflammation, proliferation, and remodeling. Upon completion of the repair process, scars form, resulting in wound healing. DFUs, a type of chronic and difficult-to-heal wound, are marked by persistent and excessive inflammatory responses. This significantly prolongs the transition from the inflammatory phase to the tissue proliferation phase, keeping the body in a state of elevated inflammation (33). The persistent inflammatory response induces the destruction of newly formed blood vessels and granulation tissue by metalloproteinases, leading to local wound edema and sustaining the inflammatory cascade, thereby affecting ulcer healing (34). Moreover, sustained hyperglycemia increases oxidative stress levels (35), downregulates growth factor receptors, and accelerates the degradation of growth factors (4), exacerbating ischemia, hypoxia, and nerve damage in diabetic wounds. In this case study, the patient presented with elevated blood glucose levels and was diagnosed with a moderate infection and medium-risk nutritional status according to the IWGDF/IDSA infection severity classification and COUNT score. The patient had a Wagner grade IV diabetic foot ulcer, accompanied by severe bone destruction, osteomyelitis, Gram-negative opportunistic pathogens, and mixed infections, indicative of a severe diabetic foot case with a poor prognosis, primarily leading to limb amputation. Consequently, we implemented systematic antimicrobial treatment, glycemic control, improved blood supply, nutritional support, and other symptomatic treatments. Resecting a single toe can significantly preserve the limb’s function with minimal impact on balance and gait (36). However, due to the low amputation level, the risk of proximal spreading of nerve and vascular lesions increases, resulting in new ulcers, infections, and non-healing wounds as postoperative complications (37). Postoperatively, the patient exhibited substantial redness, swelling, heat, and pain at the wound site, with purulent discharge along the wound edge. Cultures showed Candida albicans and Staphylococcus lugdunensis, indicating recurrent infection. The inflammatory response from foot infection and related complications can lead to high metabolic rates; infection-induced ulcer expansion or deepening worsens protein exudation, subsequently heightening the risk of malnutrition. Protein exudation reduces albumin levels, resulting in hypo-osmotic plasma, which can lead to pleural effusion, further compromising blood oxygen saturation and destabilizing vital signs. Guidance from the critical care department on antibiotic selection and pleural drainage, alongside nutritional prescriptions to correct hypoproteinemia and supplement albumin, helped the patient gradually stabilize, establishing a foundation conducive to wound healing.

TCM utilizes syndrome differentiation combined with internal and external treatments to address DFUs. This comprehensive approach enables a deeper understanding of the underlying causes and mechanisms, resulting in improved wound healing rates and symptom management (38–40). During the inflammatory phase, oral administration of the SYD and external application of MEBO are employed. The SYD contains multiple anti-inflammatory active ingredients, such as chlorogenic acid, ferulic acid, caffeic acid, glycyrrhizin, and cinnamic acid, which suppress inflammatory responses across various targets and pathways, forming the biological basis for the prescription’s anti-inflammatory properties (41–43). Additionally, it has effects on immune modulation, antioxidation, protection of vascular endothelium, regulation of angiogenesis, and improving insulin resistance, thereby facilitating diabetic foot wound healing (44–46).MEBO, an ointment preparation, interacts with necrotic tissues through hydrolysis and saponification upon adherence to the wound. It liquefies necrotic tissues, replaces surgical debridement, disrupts bacterial habitats, reduces bacterial activity, provides a moist environment for healing, and prevents bacterial invasion (47–49). Moreover, it contains amino acids, carbohydrates, and fatty acids which nourish the wound, promoting growth (50). For patients with extensive necrotic tissues and slow granulation growth, SYD and MEBO help regulate inflammation, reduce bacterial load, control infection, and transition the wound from inflammation to proliferation phase (**Table 3**). During the granuloma and re-epithelialization phase, BHD and Chinese scraping therapy combined with KFXS are used. BHD exhibits anti-inflammatory effects, inhibits secretion of inflammatory substances, prevents thromboxane synthesis, improves microcirculation, reduces inflammatory exudation, alleviates inflammation, promotes wound repair, and enhances VEGF expression, inducing neovascularization and improving hypoxia in wounds (51–56). KFXS, derived from Periplaneta americana extracts, up-regulates glutamine levels, stimulates protein synthesis, supporting cell proliferation, granulation growth, and angiogenesis, and enhances immune functions for rapid ulcer healing (57–60). Due to aged granulation tissue in this patient, Chinese scraping therapy was used to ensure drainage, remove aging tissue, and stimulate fresh growth (61). This approach accelerates granulation tissue and new blood vessel development, reduces wound size, promotes epithelial growth, and accelerates healing (**Table 3**). The treatment plan, involving Chinese medicine, vasodilators, and neurotrophic agents, improved symptoms, including numbness, pain, dry mouth, and fatigue. Follow-up calls after three months confirmed complete wound healing, allowing normal foot function and walking.

**Table 3:** Mechanism of intervention of traditional Chinese medicine compound in the treatment of DFUs.

Prescription	Composition	Main components	Medicinal efficacy	Pathways	References
Simiao Yong’an Decoction (SYD)	Radix Scrophulariae,Flos onicerae Japonicae, Radix Angelicae Sinensis, Radix et Rhizoma Glycyrrhizae	Chlorogenic acid, ferulic acid, caffeic acid, glycyrrhizin, cinnamic acid, quercetin, kaempferol and β-sitosterol	Clears heat and detoxifies, promotes blood circulation, unblocks channels	1. Regulates MAPK and TNF pathways, promotes wound angiogenesis, reduces inflammatory response: reduces serum CRP and TNF-α levels, increases bFGF, EGF, and VEGF levels; downregulates TNF-α, IL-1β; increases MAPK, VEGF, IFN-γ levels. 2. Regulates Wnt/β-catenin signaling pathway, promotes wound angiogenesis: upregulates the levels of β-catenin and Rspo proteins and mRNA, downregulates the levels of GSK-3β protein and mRNA. 3. Regulates the TLR4/NLRP3/Caspase-1 pathway to inhibit pyroptosis: downregulates TLR4, NF-KB, NLRP3, Caspase-1, IL-1β mRNA and protein expression.	(4, 33–37)
Moist exposed burn ointment (MEBO)	Rhizoma Coptidis,Radix Scutellariae, Cortex Phellodendri Chinensis,Pheretima,Pericarpium Papaveris	Quercetin, baicalein, wogonoside, palmitic acid methyl ester, and homogentisic acid	clear heat and detoxify, relieve pain, and promote tissue regeneration	1. Inhibits the AGEs-RAGE/NF-κB signaling pathway: downregulates AGEs, RAGE mRNA, and NF-κB levels. 2. Regulates the TGF-β1/Smads signaling pathway via miRNA-21, promoting fibroblast migration and proliferation: increases the expression of miRNA-21 and TGF-β1 mRNA, boosts P-Smad2 and P-Smad3 protein expression, and inhibits Smad7 protein levels. 3. Activates the PI3K/AKT/FoxO1 signaling pathway, promoting re-epithelialization: decreases the expression of PI3K, FoxO1, and Vimentin, while increasing the expression of E-cadherin and Claudin1. 4. Activates the PI3K/Akt signaling pathway, promoting angiogenesis in wound healing: upregulates PI3K, P-AKT, and VEGFR2 factors.	(38–41)
Buyang Huanwu Decoction (BHD)	Radix Astragali, Radix Angelicae Sinensis, Radix Paeoniae Rubra, Flos Carthami, Semen Persicae, Rhizoma Chuanxiong and Pheretima	Astragaloside, formononetin glucoside, ferulic acid and paeoniflorin	tonifying Qi, promoting blood circulation, and unblocking channels	1. Regulates the AGEs-RAGE pathway, inhibiting the inflammatory response: downregulates TNF-α and IL-6 expression, reduces the expression of AGEs, RAGE, ICAM-1, VCAM-1, MMP-2 proteins and mRNA. 2. Regulates the Akt/GSK3β/NRF2 pathway to exert antioxidant, anti-inflammatory, and angiogenic effects: downregulates IL-1β, IL-6, and TNF-α expression, increases the expression of HO-1, NQO-1, CAT, and VEGFA. 3. Activates the TGF-β1/Smad4 signaling to promote the maturation of new blood vessels: upregulates TGF-β1, Smad4 mRNA expression, and N-Cadherin protein expression. 4. Activates VEGFR2, regulates the PI3K/Akt signaling pathway, and induces angiogenesis: upregulates VEGF, VEGFR2 expression, increases PI3K and p-Akt protein levels.	(42–47)
KangFuXin Solution (KFXS)	American cockroach	amino acids, nucleosides, and peptides	Promotes blood circulation, nourishes Yin, and promote tissue regeneration	1. Regulates the AGEs-RAGE pathway:downregulates the expression of AGEs, RAGE, and HIF-1α proteins. 2. Promotes the expression of bFGF protein, stimulating the secretion and synthesis of growth factors: upregulates the expression of bFGF, TGF-β, and EGF proteins; increases VEGF expression and the number of new blood vessels. 3. Exerts anti-inflammatory effects by inhibiting the production of PTGS2: Downregulates the mRNA expression of PTGS2, IL-6, and TNF-α.	(48–51)

Although this case highlights the potential benefits of integrative Chinese and western medicine for diabetic foot ulcer management, its conclusions are inherently limited by the single-patient nature of a case report. Within the hierarchy of clinical evidence, such reports primarily serve as hypothesis-generating observations. Therefore, future prospective cohort or randomized controlled studies are warranted to provide higher-level evidence and verify the reproducibility of these integrative approaches.

## Conclusion

This case report demonstrates that the integration of Chinese and western medicine in treating DFUs effectively controls disease progression, improves systemic symptoms, and promotes wound repair, suggesting it is a viable treatment strategy.

## Data Availability

The datasets presented in this article are not readily available because of ethical and privacy restrictions. Requests to access the datasets should be directed to the corresponding authors.

## References

[1] Sun H Saeedi P Karuranga S Pinkepank M Ogurtsova K Duncan BB. IDF Diabetes Atlas: Global, regional and country-level diabetes prevalence estimates for 2021 and projections for 2045. Diabetes Res Clin Pract. (2022) 183:109119. doi: 10.1016/j.diabres.2021.109119, PMID: 34879977 PMC11057359

[2] Zhang P Lu J Jing Y Tang S Zhu D Bi Y. Global epidemiology of diabetic foot ulceration: a systematic review and meta-analysis. Ann Med. (2017) 49:106–16. doi: 10.1080/07853890.2016.1231932, PMID: 27585063

[3] Gu Y. Diagnosis and treatment of diabetic foot. Beijing: People’s Medical Publishing House (2016). p. 9.

[4] Patel S Srivastava S Singh MR Singh D. Mechanistic insight into diabetic wounds: Pathogenesis, molecular targets and treatment strategies to pace wound healing. BioMed Pharmacother. (2019) 112:108615. doi: 10.1016/j.biopha.2019.108615, PMID: 30784919

[5] Brem H Tomic-Canic M. Cellular and molecular basis of wound healing in diabetes. J Clin Invest. (2007) 117:1219–22. doi: 10.1172/JCI32169, PMID: 17476353 PMC1857239

[6] Li Z Guo S Yao F Zhang Y Li T. Increased ratio of serum matrix metallo Protein-ase-9 against TIMP-1 predicts poor wound healing in diabetic foot ulcers. J Diabetes Complications. (2013) 27:380–2. doi: 10.1016/j.jdiacomp.2012.12.007, PMID: 23357650

[7] Basu Mallik S Jayashree BS Shenoy RR. Epigenetic modulation of macrophage polarization- perspectives in diabetic wounds. J Diabetes Complications. (2018) 32:524–30. doi: 10.1016/j.jdiacomp.2018.01.015, PMID: 29530315

[8] Singh K Agrawal NK Gupta SK. Decreased expression of heat shock proteins may lead to compromised wound healing in type 2 diabetes mellitus patients. J Diabetes Complications. (2015) 29:578–88. doi: 10.1016/j.jdiacomp.2015.01.007, PMID: 25746357

[9] Raja JM Maturana MA Kayali S Khouzam A Efeovbokhan N. Diabetic foot ulcer: A comprehensive review of pathophysiology and management modalities. World J Clin Cases. (2023) 11:1684–93. doi: 10.12998/wjcc.v11.i8.1684, PMID: 36970004 PMC10037283

[10] Didangelos T Koliakos G Kouzi K Arsos G Kotzampassi K Tziomalos K. Accelerated healing of a diabetic foot ulcer using autologous stromal vascular fraction suspended in platelet-rich plasma. Regener Med. (2018) 13:277281. doi: 10.2217/rme-2017-0069, PMID: 29715071

[11] Everett E Mathioudakis N. Update on management of diabetic foot ulcers. Ann N Y Acad Sci. (2018) 1411:153–65. doi: 10.1111/nyas.13569, PMID: 29377202 PMC5793889

[12] Rayman G Vas P Dhatariya K Driver V Hartemann A Londahl M. Guidelines on use of interventions to enhance healing of chronic foot ulcers in diabetes (IWGDF 2019 update). Diabetes Metab Res Rev. (2020) 36Suppl 1:e3283. doi: 10.1002/dmrr.3283, PMID: 32176450

[13] Han G Ceilley R. Chronic wound healing:a review of current management and treatments. Adv Ther. (2017) 34:599–610. doi: 10.1007/s12325-017-0478-y, PMID: 28108895 PMC5350204

[14] Wang J Gao L. New progress in the treatment of chronic wounds in diabetic foot. Chin J Reparative Reconstructive Surg. (2018) 32:832–7. doi: 10.7507/1002-1892.201806058, PMID: 30129304 PMC8435952

[15] Flumignan CDQ Amaral FCF Flumignan RLG Vasconcelos VT Attie GA Daolio RM. Angioplasty and stenting for below the knee ulcers in diabetic patients: protocol for a systematic review. Syst Rev. (2018) 7:228. doi: 10.1186/s13643-018-0897-0, PMID: 30537989 PMC6290534

[16] Gao B Wang H Zhang Y Wang Y Fu H. Observation of the efficacy of modified transverse tibial bone transport in treating diabetic foot ulcers. Hainan Med. (2022) 33:2910–3. doi: 10.27282/d.cnki.gsdzu.2023.001083

[17] Rai V Moellmer R Agrawal DK. Stem cells and angiogenesis: implications and limitations in enhancing chronic diabetic foot ulcer healing. Cells. (2022) 11:2287. doi: 10.3390/cells11152287, PMID: 35892584 PMC9330772

[18] Hu XX Xiu ZZ Li GC Zhang JY Shu LJ Chen Z. Effectiveness of transverse tibial bone transport in treatment of diabetic foot ulcer: A systematic review and meta-analysis. Front Endocrinol (Lausanne). (2023) 13:1095361. doi: 10.3389/fendo.2022.1095361, PMID: 36686461 PMC9846025

[19] Hua Q Zhang Y Wan C Zhang D Xie Q Zhu Y. Chinese association of orthopaedic surgeons (CAOS) clinical guideline for the treatment of diabetic foot ulcers using tibial cortex transverse transport technique (Version 2020). J Orthop Translat. (2020) 25:11–6. doi: 10.1016/j.jot.2020.05.003

[20] Chen Y Kuang X Zhou J Zhen P Zeng Z Lin Z. Proximal tibial cortex transverse distraction facilitating healing and limb salvage in severe and recalcitrant diabetic foot ulcers. Clin Orthop Relat Res. (2020) 478:836–51. doi: 10.1097/CORR.0000000000001075, PMID: 31794478 PMC7282570

[21] Sala F Thabet AM Castelli F Miller AN Capitani D Lovisetti G. Bone transport for postinfectious segmental tibial bone defects with a combined ilizarov/taylor spatial frame technique. J Orthop Trauma. (2011) 25:162–8. doi: 10.1097/BOT.0b013e3181e5e160, PMID: 21321507

[22] Paley D Maar DC. Ilizarov bone transport treatment for tibial defects. J Orthop Trauma. (2000) 14:76–85. doi: 10.1097/00005131-200002000-00002, PMID: 10716377

[23] Zhang J Zhou R Xiang C Jia Q Wu H Yang H. Huangbai liniment accelerated wound healing by activating Nrf2 signaling in diabetes. Oxid Med Cell Longev. (2020) 2020:4951820. doi: 10.1155/2020/4951820, PMID: 32566084 PMC7271242

[24] Yang WF Wei Q Tong Q Cui K He G Lin L. Traditional chinese medicine Tanreqing inhibits quorum sensing systems in pseudomonas aeruginosa. Front Microbiol. (2020) 11:517462. doi: 10.3389/fmicb.2020.517462, PMID: 33391189 PMC7775676

[25] He Q Yu W Wang M Su X Yao B Yang Y. Efficacy of external application of Sān Huáng Xuè Jié formula in treating the pattern of damp-heat toxin excess in diabetic foot and its effect on infected wounds. J Hebei Traditional Chin Med. (2023) 38:43–6. doi: 10.16370/j.cnki.13-1214/r.2023.04.012

[26] Zhang D Cao J Ju S. Effect of xuejieShengji ointment on expression of VEGF and PCNA in skin ulcer of diabetic rats. Chin J Integr Med Surg. (2018) 24:737–42. doi: 10.3969/j.issn.1007-6948.2018.06.013

[27] Shen Y Li Y Song Z Li Y Zhao Y Zhang H. Effects and mechanism of Maiguan Fukang Tablets combined with Tangbi Waixi Prescription on diabetic angiopathy. Chin J Integr Med Surg. (2023) 29:440–4. doi: 10.3969/j.issn.1007-6948.2023.04.003

[28] Senneville Éric Albalawi Z Asten SAv Abbas ZG Allison G Aragón-Sánchez J. IWGDF/IDSA guidelines on the diagnosis and treatment of diabetes-related foot infections (IWGDF/IDSA 2023). Clin Infect Dis. (2024) 79(1):286. doi: 10.1093/cid/ciad527, PMID: 37779457

[29] Falanga V. Wound healing and its impairment in the diabetic foot. Lancet. (2005) 366:1736–43. doi: 10.1016/S0140-6736(05)67700-8, PMID: 16291068

[30] Zhang Z Zhang Y Yang D Luo Y Luo Y Ru Y. Characterisation of key biomarkers in diabetic ulcers via systems bioinformatics. Int Wound J. (2023) 20:529–42. doi: 10.1111/iwj.13900, PMID: 36181454 PMC9885479

[31] Lavery LA Suludere MA Attinger CE Malone M Kang GE Crisologo PA. WHS (Wound Healing Society) guidelines update: Diabetic foot ulcer treatment guidelines. Wound Repair Regen. (2024) 32:34–46. doi: 10.1111/wrr.13133, PMID: 38032324

[32] Chen P Vilorio NC Dhatariya K Jeffcoate W Lobmann R McIntosh C. Guidelines on interventions to enhance healing of foot ulcers in people with diabetes (IWGDF 2023 update). Diabetes Metab Res Rev. (2024) 40:e3644. doi: 10.1002/dmrr.3644, PMID: 37232034

[33] Smigiel KS Parks WC. Macrophages, wound healing, and fibrosis: recent insights. Curr Rheumatol Rep. (2018) 20:17. doi: 10.1007/s11926-018-0725-5, PMID: 29550962

[34] Rehak L Giurato L Meloni M Panunzi A Manti GM Uccioli L. The immune-centric revolution in the diabetic foot: monocytes and lymphocytes role in wound healing and tissue regeneration-a narrative review. J Clin Med. (2022) 11:889. doi: 10.3390/jcm11030889, PMID: 35160339 PMC8836882

[35] Afonso AC Oliveira D Saavedra MJ Borges A Simões M. Biofilms in diabetic foot ulcers: impact, risk factors and control strategies. Int J Mol Sci. (2021) 22:8278. doi: 10.3390/ijms22158278, PMID: 34361044 PMC8347492

[36] Chou SW Cheng HY Chen JH Ju YY Lin YC. The role of the great toe inbalance performance. J Orthop Res. (2009) 27:549–54. doi: 10.1002/jor.20661, PMID: 18932241

[37] Berceli SA Brown JE Irwin PB Ozaki CK. Clinical outcomes after closed,staged and open forefoot amputations. J Vasc Surg. (2006) 44:347–52. doi: 10.1016/j.jvs.2006.04.043, PMID: 16890866

[38] Liu H Zhang C Qiu T Chen L Wang Y Dong Y. Meta-analysis on the efficacy and safety of Chinese herbal compound dressing in treating diabetic foot ulcers. J Guangzhou Univ Traditional Chin Med. (2023) 40:1819–25. doi: 10.13359/j.cnki.gzxbtcm.2023.07.037

[39] Li H Lu Z Guo X Niu C. Clinical effect of erhuang decoction in treatment of type 2 diabetes mellitus with qi and yin deficiency and blood stasis syndrome. Chin J Traditional Chin Med. (2021) 39:238–40. doi: 10.13193/j.issn.1673-7717.2021.01.058

[40] Wang Y. Application effect analysis of Yì Qì Yǎng Yīn Jiàng Táng Yïn with modifications in treating Qi-Yin deficiency type 2 diabetes patients. China Pract Med. (2023) 18:136–8. doi: 10.14163/j.cnki.11-5547/r.2023.06.041

[41] Zhen L Wang M Chen Z Li X. Research progress on simiao yong'an decoction. Chin Patent Medicinal Formula. (2019) 41:1365–70. doi: 10.3969/j.issn.1001-1528.2019.06.033

[42] Zhao Y Liu M Zhang Y Wang B Zhang Y Hao Q. Effect of Simiao Yong'an Decoction on the expression of Wnt/β-catenin signaling pathway in diabetic ulcer rats. Chin J Integrated Traditional Western Med. (2017) 37:79–85. doi: 10.7661/CJIM.2017.01.0079, PMID: 30695430

[43] Yuan L Wei J Zhao Y Wang G Zhang Y Zhang Y. Promoting healing mechanism of Simiao Yong’an Decoction in diabetic ulcer rats from the perspective of glycogen synthase kinase-3β intervention in inflammatory response. Chin J Traditional Chin Med. (2023) 38:826–30.

[44] Chen X Li M Wang Z Zhu M Zhang Z Wei Z. Application analysis of simiao yong’an decoction promoting wound angiogenesis in patients with diabetic foot ulcer. Chin J Traditional Chin Med. (2022) 40:62–4. doi: 10.13193/j.issn.1673-7717.2022.03.015

[45] Yu N Song N Wang Y Chen S Lv M Yang G. Study on mechanism of simiao yong’an decoction inhibiting pyroptosis pathway TLR4/NLRP3/caspase-1 to prevent and treat atherosclerosis. Chin J Traditional Chin Med. (2021) 39:199–203+279. doi: 10.13193/j.issn.1673-7717.2021.08.048

[46] Zhao Y Wang L Liao H Kuang G Zhou S Zhao Q. Exploring the mechanism of Simiao Yong'an Decoction in treating diabetic foot based on network pharmacology and molecular docking. Hunan J Traditional Chin Med. (2023) 39:166–73. doi: 10.16808/j.cnki.issn1003-7705.2023.08.039

[47] Li J Wang L Zhang C Di J Li H. Effect of MEBO on advanced glycosylation end products and their Receptors in wound tissue of rats with diabetic skin ulcer. Chin Gen Pract. (2016) 19:3153–9. doi: 10.3969/j.issn.1007-9572.2016.26.006

[48] Li J Liang B Chen Z Liu M Wang L Lu W. Effects of MEBO on miRNA-21 and its target TGF-β1/Smads signaling pathway in diabetic wound tissues of rats. Chin J Gerontology. (2024) 44:4448–52. doi: 10.3969/j.issn.1005-9202.2024.18.027

[49] Tong D Ma Z Jiang Y Zuo Y Tang Q. Discussion on the role of Moist Burn Ointment in the EMT process of diabetic rat wounds based on the PI3K/AKT/FoxO1 signaling pathway. Lishizhen Med Materia Med Res. (2024) 35:2351–61.

[50] Teh YM Mualif SA Lim SK. A comprehensive insight into autophagy and its potential signaling pathways as a therapeutic target in podocyte injury. Int J Biochem Cell Biol. (2022) 143:106153. doi: 10.1016/j.biocel.2021.106153, PMID: 34974186

[51] Shi S. Study on the therapeutic effect of Buyang Huanwu Decoction via regulating AGEs-RAGE signaling pathway on diabetic wounds. Shandong Univ Traditional Chin Med. (2023). doi: 10.27282/d.cnki.gsdzu.2023.001083

[52] Bao XY Deng LH Huang ZJ Daror AS Wang ZH Jin WJ. Buyang huanwu decoction enhances revascularization via akt/GSK3β/NRF2 pathway in diabetic hindlimb ischemia. Oxid Med Cell Longev. (2021) 2021:1470829. doi: 10.1155/2021/1470829, PMID: 34900083 PMC8664534

[53] Yang L Lu B Pang X Li H Shu L Sun Y. Exploration of the mechanism of Buyang Huanwu Decoction promoting new blood vessel maturation under hypoxic conditions based on TGF-β1/Smad4 signaling. Chin J Exp Formulas. (2018) 24:114–9. doi: 10.13422/j.cnki.syfjx.2018030114

[54] Cui HJ Yang AL Zhou HJ Wang C Luo JK Lin Y. Buyang Huanwu decoction promotes angiogenesis via vascular endothelial growth factor receptor-2 activation through the PI3K/Akt pathway in a mouse model of intracerebral hemorrhage. BMC Complement Altern Med. (2015) 15:91. doi: 10.1186/s12906-015-0605-8, PMID: 25886469 PMC4381446

[55] Wei Q Zhao X. Clinical observation on Buyang Huanwu decoction combined with VSD technology in treating diabetic foot. Clin Res Traditional Chin Med. (2015) 7:115–6.

[56] Liu W Wang J Gui D. Current situation and prospects of Buyang Huanwu decoction combined with interventional therapy in treating diabetic foot. China Med Herald. (2019) 16:29–32.

[57] Xu J Tong S Shu G Zhou Y Dai X Wang B. Impact of KangFuXin Solution on the expression levels of AGEs, RAGE, and HIF-1 in non-healing wounds in diabetes. Guangdong Med. (2024) 45:231–5. doi: 10.13820/j.cnki.gdyx.20232605

[58] Wang F Luo Y Geng F Shen Y Wang Y Song J. Research on the mechanism of the accelerated healing of the pressure ulcer by Kangfuxin liquids on the mice. Huaxi J Pharm Sci. (2017) 32:260–3. doi: 10.13375/j.cnki.wcjps.2017.03.012

[59] Hu Y Xiong Q Li X Shi W. Effects of different concentrations of Kangfuxin solution on wound repair and VEGF expression in chronic ulcer. Chin Med Aesthetics. (2021) 11:43–6. doi: 10.19593/j.issn.2095-0721.2021.06.012

[60] Tang Y Zhang Y Li G Li Q. Anti-inflammatory effect and mechanism of KangFuXin Solution in macrophage inflammatory response. Shandong Med. (2016) 56:13–5.

[61] Fu R Wu Y Xie B Zhen C. Clinical effect of applying scraping therapy based on the “removal of necrotic tissue to promote muscle growth” theory in treating post-horseshoe-type perianal abscess. Inner Mongolia J Traditional Chin Med. (2024) 43:135–6. doi: 10.16040/j.cnki.cn15-1101.2024.03.082

